# Modeling of coupled motion and growth interaction of equiaxed dendritic crystals in a binary alloy during solidification

**DOI:** 10.1038/srep45770

**Published:** 2017-03-31

**Authors:** Xin Bo Qi, Yun Chen, Xiu Hong Kang, Dian Zhong Li, Tong Zhao Gong

**Affiliations:** 1 Shenyang National Laboratory for Materials Science, Institute of Metal Research, Chinese Academy of Sciences, Shenyang, Liaoning, 110016, P. R. China

## Abstract

Motion of growing dendrites is a common phenomenon during solidification but often neglected in numerical simulations because of the complicate underlying multiphysics. Here a phase-field model incorporating dendrite-melt two-phase flow is proposed for simulating the dynamically interacted process. The proposed model circumvents complexity to resolve dendritic growth, natural convection and solid motion simultaneously. Simulations are performed for single and multiple dendritic growth of an Al-based alloy in a gravity environment. Computing results of an isolated dendrite settling down in the convective supersaturated melt shows that solid motion is able to overwhelm solutal convection and causes a rather different growth morphology from the stationary dendrite that considers natural convection alone. The simulated tip growth dynamics are correlated with a modified boundary layer model in the presence of melt flow, which well accounts for the variation of tip velocity with flow direction. Polycrystalline simulations reveal that the motion of dendrites accelerates the occurrence of growth impingement which causes the behaviors of multiple dendrites are distinct from that of single dendrite, including growth dynamics, morphology evolution and movement path. These polycrystalline simulations provide a primary understanding of the sedimentation of crystals and resulting chemical homogeneity in industrial ingots.

Dendritic crystal growing from melt often undergoes a variety of physical phenomena and their interactions. One important phenomenon that has been neglected for a long term is the movement of growing crystal. In both experiments and practices, motion of crystal occurs inevitably when the melt is solidified under gravity environment^1^,^2^. The motion of solid mainly stems from two forces: the gravity-driven moving force induced by density difference between the crystallizing dendrites and the melt, and external forces exerted by specific industrial processing, such as mechanical vibration, electro-magnetic stirring. Solid motion is a crucial factor for microstructure formation and thereby the properties of castings. Dendrites move relative to the melt, not only altering the distribution of temperature and concentration fields, but also in turn affecting the shapes and growth dynamics^3^. For instance, it is generally realized that the motion of equiaxed dendrites is responsible for the cone-shape negative segregation in the bottom of large steel ingot^4^,^5^,^6^.

However, despite the fact that abundant experimental, theoretical and numerical studies concerning dendrite growth under diffusion and convection have been performed during the past several decades, direct studies involving dendrite motion are rarely reported. Comprehensive understanding of its effect on dendritic growth still remains a pending issue. This is because handling the coupled dynamical behaviors between moving solid, flowing melt and growing dendrites presents a significant challenge. Experimentally, the conventional method by postmortem analysis of the sample only provides a frozen solid metallography, which is limited to confirm whether crystals moving or not during solidification, let alone the analysis of its influence on crystal morphology. Direct *in situ* observation is a privileged choice of studying the movement of dendrite but always limited for special materials or experimental configurations. Appolaire *et al*. studied the influence of movement of equiaxed dendrites on their growth kinetics using NH_4_Cl-H_2_O solution^7^,^8^. The results showed that the settling of dendrite greatly promoted the growth kinetics even for weak settling velocities. Badillo *et al*. carried out several experiments to measure tip velocities of six primary dendrite arms of cubic SCN-acetone alloy and investigated the effects of settling speed and inclination angle on growth dynamic in details^9^. Their experimental results agree well with the boundary layer model derived from previous works^10^,^11^. However, these experimental investigations are unable to clarify the coupled effect of thermosolutal convection on the dendritic growth process.

In order to exactly recover what happens in solidification simulations, the underlying multiphysics, including melt convection, solid movement, thermal and solutal diffusion, and the interplays among these effects are necessitated to be incorporated into the numerical models. Do-Quang and Amberg proposed a combination of phase-field model with a fictitious domain method to simulate a settling dendrite due to gravity^12^. The translation and rotation of a dendrite were successfully represented in the simulation. Medvedev *et al*. developed a combination of phase-field method with Lattice Boltzmann method (PFLB in abbreviation) to simulate mobile dendrites in a flow^13^. Recently, Rojas *et al*. and Takaki *et al*. also used the PFLB method to simulate motion and growth of a dendrite^14^,^15^. Both methods consist of two parts: the phase-field model is implemented to calculate the growth of dendrites, while the fictitious domain method or Lattice Boltzmann method is employed to compute the motion of liquid and solid. In practice, of these methods in calculating solid moving velocity, precise tracking of the solid and liquid interface is required to calculate the total force and torque acting on the solid through integrating viscous stress and pressure across the interface. Given the complex morphology of dendrite, these approaches encounter a delicate numerical treatment of the momentum exchange between solid and liquid, which is in particular intricate when simulating polycrystalline growth. Besides the overhead in keeping track of moving interfaces, these methods lose efficiency when interfaces undergo topological changes such as merging of two neighboring dendrites with a small misorientation, detaching of sidebranches and cracking of dendrite arms^16^.

In this paper, an optional model for dendritic growth and motion is presented that permits the simulation without calculating the force acting on the solid by melt flow and handles topological changes conveniently. This is accomplished by extending the conventional phase-field model coupled with Navier-Stokes equations for two-phase flow. Phase-field model has been validated to offer a straightforward route for conducting two-phase flow simulations successfully^17^,^18^,^19^. A wide range of two-phase flow problems including Rayleigh–Taylor instability^20^, Hele-Shaw flow^21^, bubble rise and coalescence^17^, Marangoni flow^22^ are well solved by this method. Moreover, since all the governing equations are partial differential equations which can be solved in a unified framework, significant simplification in the code structure can be achieved in respect of computation. Through comparing the calculations with several typical solid-liquid two-phase flow examples, the model is verified and applied to simulate motion of dendrites with melt convection in Al-4wt.% Cu alloy solidification. Its effects on the dendritic growth dynamics are also illustrated and clarified.

## Model Description

The phase-field model proposed by Karma for alloy solidification^23^ and the procedures by Warren *et al*. for polycrystalline materials^24^ are combined to simulate dendritic alloy solidification from a supersaturated liquid melt. The phase-field parameter *ϕ* is used to indicate the distribution of the phases, with *ϕ* = ±1 inside the bulk solid and liquid, respectively. Similar to previous work for solid motion^12^,^13^,^14^,^15^,^25^, the advection term 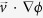 is added into the evolution equation in order to update the position of solid phases, where 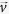 is the liquid flow vector. Then the phase-field equation yields to


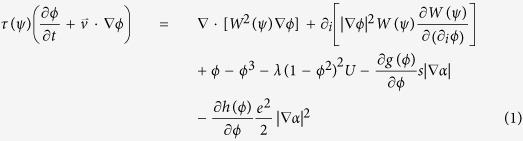


where 
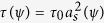
 and *W(ψ*) = *W*_0_*a*_*s*_*(ψ*) are relaxation time and interface thickness with *ψ* =* α*_*n*_ −* α*, respectively. The symbol *i* stands for *x* and *y* axis, respectively, and it obeys Einstein summation convention. The anisotropy angle *α*_*n*_ is the angle between interface normal and *x* coordinate axis and *α* is crystallographic orientation. The simplified four-fold anisotropy function *a*_*s*_*(ψ*) = 1 +* ε* cos4*ψ* is adopted with *ε* the anisotropy strength of surface energy. *λ* = 0.8839*W*_0_/*d*_0_ is the coupling coefficient between phase field *ϕ* and dimensionless concentration field *U. d*_0_ = Γ/[|*m*|*c*_0_(1 − *k*)] is chemical capillary length with *m* liquidus slope and *k* solute partition coefficient, *Γ* Gibbs-Thomson coefficient, *c*_0_ initial concentration. The choices of *τ, W*, and *λ*, same as that of Karma^23^ lead to zero interface kinetics, hence Eq. (1) only focuses on slow solidification. The last two terms on the right-hand side account for the effect of gradient of crystal orientation angle on the phase field for multiple grains. *g(ϕ*) = [(1 + *ϕ*)/2]^2^ is a monotone function in the interval [−1, 1], and *h(ϕ*) takes the same form as *g(ϕ*). And *s* = *W*_0_/1.06 and *e* = *W*_0_/1.875 are angle gradient coefficients that can be related to grain boundary properties^25^. The governing equation for solute conservation is modified from the diffusion-limited model proposed by Karma^23^ to account for the convective transport of solute, i.e.


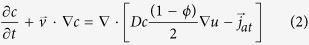


where *c* is solute concentration and. 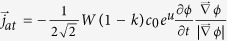 is a solute flux from the solid to the liquid along the direction normal to the interface to eliminate the unphysical effects induced by the finite thin interface. *u* is the dimensionless variable for *c* which is given as 
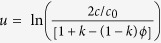
. The variable *u* measures the departure of the chemical potential from its equilibrium value for a flat interface at temperature *T*_0_ where the liquidus concentration is equal to *c*_0_. To circumvent numerical computations of exponential and logarithm functions, a new variable *U* = (*e*^*u*^ −1)/(1 − *k*) is introduced. Replacing the concentration *c* in Eq. (2) with dimensionless *U*, the solute conservation equation is rewritten as


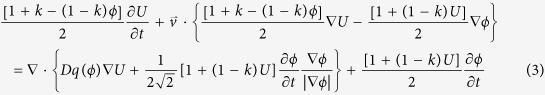


where *D* is the solute diffusivity in liquid. Here, it may be noticed that Eq. (3) has already considered the solute transport by the movement of solidified dendrites. For polycrystalline solidification, the crystallographic orientation evolution which includes crystal rotation reads^24^,^25^,^26^


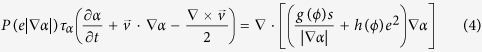


where *τ*_*α*_ = 0.1*τ*_0_ is a kinetic scaling factor for orientation field and *P(e*|∇*α*|) is an inverse mobility function, *P(w*) = 1 − (1 + *μ/e*)exp(−*βw*), where *μ* = 10^3^*W*_0_ and *β* = 10^5^ are kinetic coefficients that respectively control the relaxation time in the bulk and grain boundary regions^24^. The curl term on the left hand side of Eq. (4) represents the torque force induced by melt flow on the rotation of a crystal. In the model, the solid is not artificially assumed rigid but with high viscosity. This assumption refers to the schemes of conventional diffusive interface model for two incompressible viscosity-matched fluids^17^,^18^. The advantage of this method is that the motion of liquid and solid can be calculated simultaneously within one set of governing equations. Phase-field simulations using this model for incompressible flow of two phases of which the density ratio is close to 1000 and viscosity ratio is close to 70 demonstrate that the model is valid for flow problems with large density and viscosity ratios^19^. This conclusion is confirmed by further simulations^27^ where the viscosity and density ratios of two phases are 100 and 1000, respectively. It can be roughly extrapolated that the phase-field model can handle two-phase flow problems with large property contrast. In this work, the liquid is assumed Newtonian and incompressible while the convection is laminar. With the help of the concept of phase-field model for two liquid phases flow, the unified flow equations in the entire domain is therefore described as


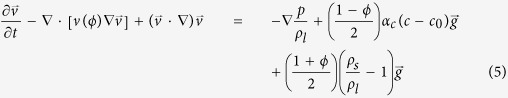


where *p* is the pressure, 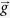 is the gravity acceleration vector, *α*_*c*_ is the solutal expansion coefficient, *c* is the solute concentration, *ρ*_*s*_ and *ρ*_*l*_ are the solid density and liquid density, respectively. Here the way to deal with density variation is to use the classical Boussinesq approximation^17^,^28^. The second term on the right hand of Eq. (5) is responsible for buoyant force in melt induced by variation of solute concentration, while the third term accounts for gravitational force due to density difference between solid and liquid. *ν(ϕ*) = *ν*_*s*_(1 + *ϕ*)/2 + *ν*_*l*_(1 − *ϕ*)/2 is a linear average of the kinematic viscosity of solid *ν*_*s*_ and liquid *ν*_*l*_. The mass conservation equation is given as


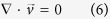


In order to increase computational efficiency, the adaptive finite element method is adopted, where meshes can be dynamically coarsened or refined according to a local error indicator^29^. The numerical implementation is based on the open-source finite element package AFEPack^30^. A series of numerical tests are presented in the Supplementary Materials, including the rising of a circular particle, rotation of a growing dendrite at a constant angular velocity and single crystal in a forced shear flow. These numerical tests verify the accuracy of the proposed model in handling dendrite-melt two phase flow and the choice of interface width parameter, *W*_*0*_ (see Figs S1–S5 and corresponding analysis in the Supplementary Note).

## Results and Discussion

The model is then applied to simulate the dendritic solidification under gravity environment. The selected alloy system for simulations is Al-4wt.% Cu, of which the physico-chemical parameters as well as the applied solidification parameters are listed in Table 1. It should be noted that surface energy anisotropy strength *ε*_4_ is set to be 0.05 for expediency to make the dendrites grow more quickly rather than 0.0094~0.0106 obtained by experimental measurements by Liu *et al*.^31^. Since the solidification velocity is slow, the phase-diagram is in local-equilibrium, in accordance with zero kinetic undercooling assumption in the phase-field governing equation. The dendrite grows under an isothermal (i.e. a constant super-saturation) condition. Hence parameters, including the liquidus slope and solute partition coefficient, are constants which are extracted from the phase-diagram of Al-Cu alloy at a given undercooling.

### Simulations of a dendrite settling down in a gravity environment

A free crystal growth is always of importance to single out the underlying evolution dynamics. Hence, the potential of the built model is first addressed via simulation of an equiaxed dendritic crystal growth in consideration of solutal convection and solid phase motion. This simple situation permits a clear recognition of these two effects on equiaxed crystal growth.

**Figure 1:**
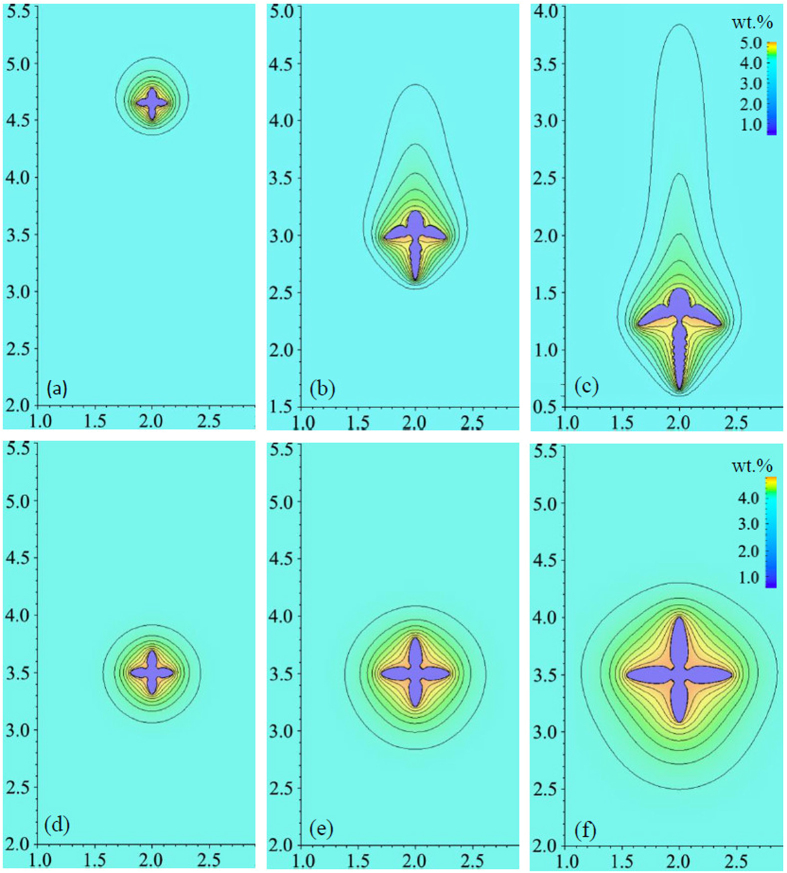
The snapshots of simulated dendritic morphology and solute concentration. (**a–c**) Evolution of mobile dendrite at solidification time *t* =  0.32 s, 0.85s, 1.22 s; (**d–f**) stationary dendrite only considering solutal convection at *t* = 0.53 s, 1.06 s, 1.96 s. The interval between concentration isolines is 0.1 wt.% with outmost line equal to 4.05wt.%. The dimensionless coordinate values have been scaled by 1000.

**Figure 2:**
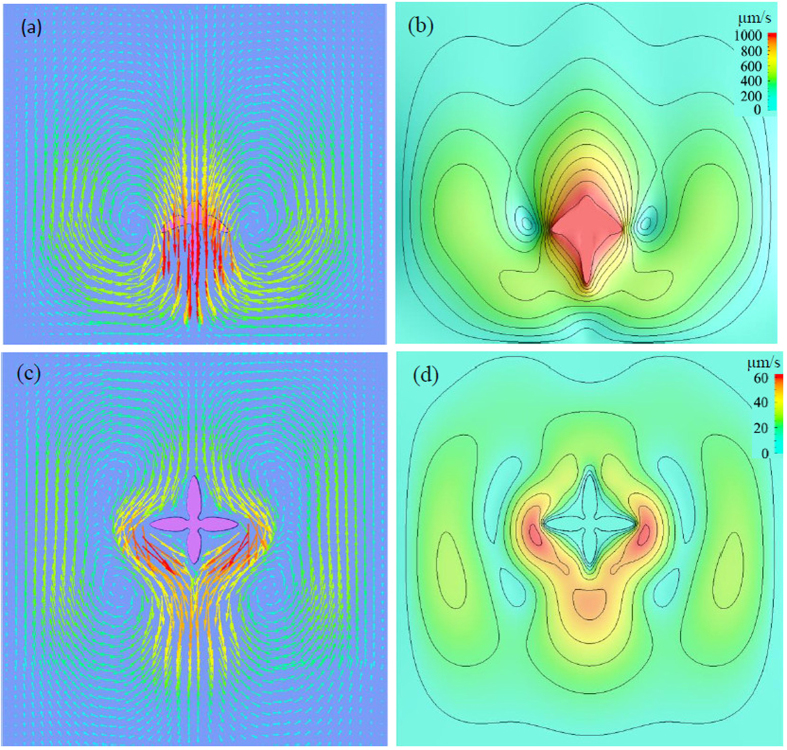
Flow field and its contour for the two cases (**a, b**) with consideration of settling motion of solid phase at *t* = 1.22 s; the interval between velocity isolines is 100 μm/s. (**c, d**) without consideration of settling motion of solid phase at *t* = 1.96 s; the interval between velocity isolines is 10 μm/s.

The simulation is conducted in a domain with size of 4000 × 7000 (856 μm × 1498 μm). Zero-flux Neumann condition is imposed on the boundaries of the computational domain for both phase-field and solute concentration equations, which is always valid in the following simulations unless otherwise stated. No-slip boundary condition is applied to all the walls of the domain for the Navier-Stokes equations. A round seed nucleates in the upper part of the domain to guarantee the sufficient long path for the dendrite to move downwards in a gravity environment. It may be noticed that unlike the Al-based alloy with higher concentration of cooper, i.e. Al-10wt.% Cu or above, the solid density for Al-4wt.% Cu alloy is larger than liquid and hence dendrite settling is expected^2^. This phenomena is convinced in the *in situ* and real-time observed experiment of Al-4wt.% alloy^32^, where some dendrites moved downwards slightly. Figure 1(a–c) shows the snapshots of dendritic morphology and concentration contours of the dendrite evolving with solidification time. Obviously, due to gravity effects the dendrite settles down as it grows from the saturated melt. What’s more, to keep local equilibrium, the heavier solute Cu is rejected from solid into adjacent liquid, and results into a downward flow along the solid-liquid interface. This solutal convection contributes another force to the movement, pushing the dendrite settling faster. Therefore, solid sedimentation is not only caused by the gravity force induced by density variation between two phases, but also is accelerated by solutal convection. For purpose of comparison, the growth of a stationary dendrite which only considers the solutal convection effect is provided in Fig. 1(d–f). The corresponding flow field and velocity magnitude of these two cases are shown in Fig. 2. Overall, settling motion of the solid exerts a large interfacial drag force on the liquid and pulls the liquid downwards, apparently contrast to the weak flow for stationary dendrite that considers solutal convection alone. Because of the symmetry pattern of the melt flow along the vertical crystal axis, the mobile dendrite does not exhibit obvious rotation during settling. As a further validity to the two-phase flow model, the velocity in the solid region appears as monolithic flow, which is shown by the isolines of magnitude in Fig. 2(b).

At the beginning of solidification for both cases, the melt flow is weak, hence, transportation of solute by convection is limited and crystal growth is controlled by diffusion. This is reflected by the symmetry shape of solute isolines around the equiaxed crystal in Fig. 1(a,d). However, as solid-liquid interface progresses further into melt, the symmetry breaks down in both cases, featured by the compressed (and stretched) isolines around the vertically growing arms, as shown in Fig. 1(b,c,e,f). In the case of considering solid motion, the largest solutal gradient lies ahead of the downward growing tip, totally contrary to the situation only considering solutal convection. This is because although the rejected solute sinks down from the upper arm to the lower one, settling motion causes the tip front of the downward growing arm penetrating into the liquid, which shortens the thickness of solute boundary layer dramatically. The different solute profile surrounding the dendrite thus results in pronounced different morphology between these two cases, and here is hence evident that the motion of dendrite is capable of overwhelming the effects induced by solutal convection. However, this situation would change as Δ*ρ*_*sl*_/Δ*ρ*_*c*_ approaches to unit with Δ*ρ*_*sl*_ the solid-liquid density difference and Δ*ρ*_*c*_ the density resulting from solutal expansion. Presently, the ratio approximates to 11.

**Figure 3:**
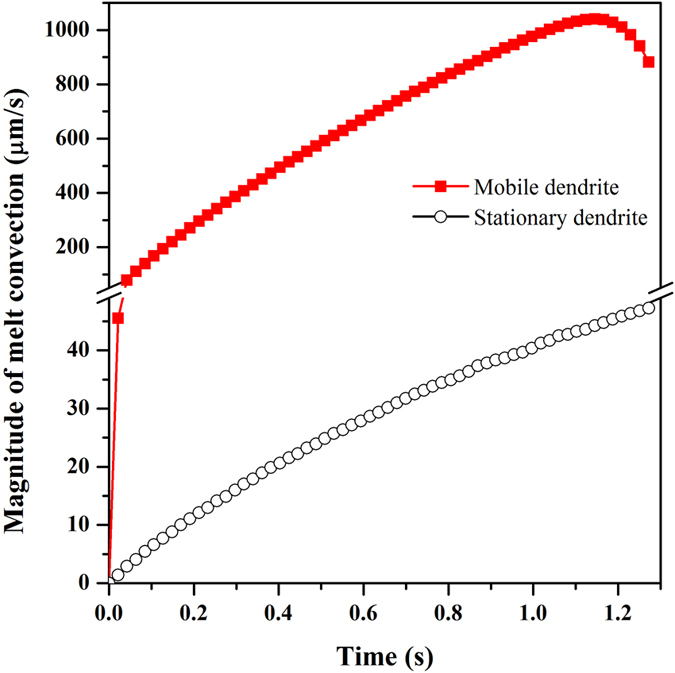
Time evolution of convection strength in two cases shown in Fig. 1(a–c) and (d–f)

Figure 3 shows the time evolution of maximum melt flow velocity which keeps increasing for the two cases. The increment of melt flow driven by solutal convection is ascribed to more solute rejected into the melt ahead of the interface, while for the dendrite considering solid motion, the larger increase amplitude should be attributed mostly to the dendrite growing into larger size. According to the Stokes’ law, the floating or settling velocity of a particle in liquid is proportional to the square of the particle geometrical size. When the dendrite moving closer to the bottom wall of the domain, the viscosity boundary layer of convection is restricted, which hence weakens the dragging flow. Thus, the melt flow velocity reaches a peak value and then decreases at the end of simulation. After the initial rapid increase and before the decrease from the peak, the flow velocity considering solid motion keeps around 24 times of that only considering solutal convection. And the corresponding fluid Reynolds number 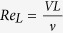 varies from initial 0 to the end 3. Here in the calculation of *Re*_*L*_, length of the computational domain is chosen as the characteristic length *L*, and the settling velocity of the dendrite is selected as the characteristic velocity *V*. Apparently, the type of flow is laminar, consistent with the assumption.

**Figure 4:**
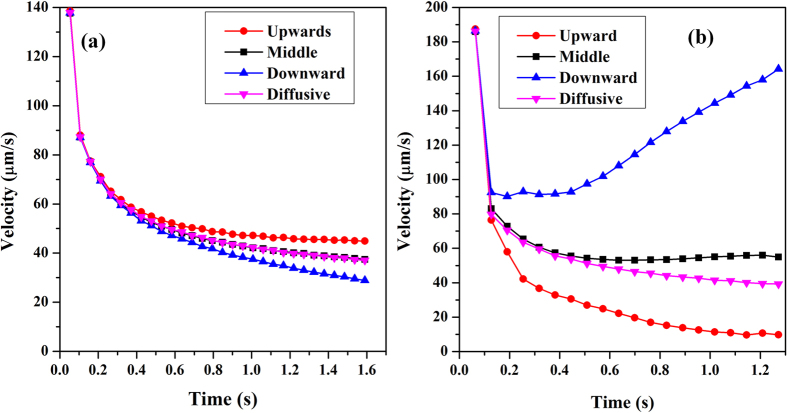
Time evolution of the tip growth velocities, (**a**) stationary dendrite; (**b**) mobile dendrite. The tip growth velocity of diffusion-controlled dendrite is plotted as well.

As is described above, when the motion of dendrite is taken into account, the flow differs a lot from that only driven by solutal buoyancy. Therefore, dendritic growth dynamics certainly varies with this alteration of flow. Detailed dendritic tip growth velocity in two cases are quantitatively compared in Fig. 4. At the early stage of solidification, the melt convection in both cases is so weak that its influence on dendrite growth is not obvious as indicated by the coincide curves. Gradually, the difference between the three tips becomes more and more noticeable. When only considering the convection driven by solutal buoyancy, the upward tip grows much faster than downward and middle tips. The growth rate of upward tip is about 1.5 times larger than that of downward tip and 1.2 times larger than that of diffusion-controlled steady-state growth. Nevertheless, when taking account of solid motion, the growth of vertical tips presents rather different behaviors. The downward tip grows in a much higher velocity than that of upward and middle tips. Owing to continuous increase of settling speed of the dendrite, the growth rate of the downward tip increases gradually after the initial rapid decrease rather than reaches a plateau. Whereas, the growth of upward tip is heavily constrained. Because of the strong convection, the dendrite growth is controlled by flow, as denoted by the significant departure of growth rate from the diffusion-controlled steady-state dendrite. At the end of simulation, the downward tip growth velocity is around 4.0 times higher than the diffusion-controlled velocity.

Although the relative relation among three tip velocities in two cases seems totally opposite, the growth follows the same underlying physics, i.e. the speed strongly depends on the solute gradient and the tip undercooling. For the stationary dendrite, melt convection compresses the solute boundary layer and promotes the upstream tip growth by taking rejected solute away adequately, while it loosens the concentration isolines adjacent to the downstream arm and retards its tip growth by accumulating solute adjacent to the tip front. However, though the pattern of melt convection is similar to that of stationary dendrite, the solute gradient is steeper ahead of the downward tip for the mobile dendrite because of the fast settlement which narrows the distance between the tip interface and bulk melt. This leads to the tip interface contacting the “fresh” melt closely and thereby a high tip undercooling.

**Figure 5:**
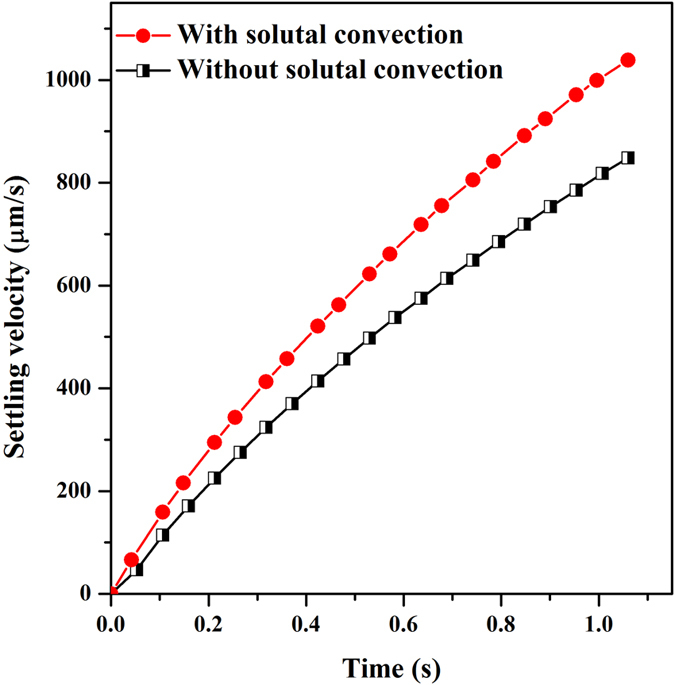
The effect of solutal convection on the settling velocity of dendrite.

Since the solutal convection which descends along the solid-liquid interface accelerates the settlement of dendrites through exerting a downward force, simulation of mobile dendrite free from solutal convection is performed to clarify this effect on settlement for convenience. The instantaneous settling velocities of mobile dendrite with and without consideration of solutal convection are plotted in Fig. 5. Overall, the settling velocity increases in a linear fashion with time for both cases whose evolution feature is in the same manner as the result obtained by Badillo *et al*.^9^. When solutal convection is taken into consideration, the dendrite settles much faster. For the present simulation configuration, the settling velocity increases up to 20% at the late solidification stage. Hence, it demonstrates that the interaction between solutal convection and solid motion is in a coupled behavior. When convective flow is in line with the moving direction of solid, both convection and movement will be strengthened, while in an opposite direction, their dynamics will be weakened. This phenomenon is further evidenced and analyzed in the following simulation of polycrystalline condition.

### Correlating the simulations with an analytical theory

The influence of dendrite settlement on growth dynamics of transparent alloy has been studied experimentally by Badillo *et al*.^9^. Based on the experimental data, the boundary layer model which accounts for the effects of thermosolutal convection on free dendritic growth of alloys^10^,^11^,^33^,^34^ is modified to address the flow angle dependence. The modified model is found to predict well the variation in the tip velocity due to crystal rotation and settling speed change^9^. Here the phase-field simulation of single mobile dendrite is examined by the solution of the analytical model. It should be noted that the 3D Ivantsov paraboloid solution rather than the 2D parabolic equation is adopted in the analytical model here. According to the study by McFadden and Browne^35^, dendrite tip growth rate calculated by 3D Ivantsov solution can be more than 10 times larger than that by 2D results at slightly high undercooling. However, the numerical results by phase-field model for the tip velocity are different for 3D versus 2D by a factor of only 1.76~2.0^36^,^37^. The mismatch ratio between phase-field simulation and analytical solution leads to the comparison intricacy. As the 3D analytical solution predicts better the experimental data, hence the modified 3D Ivantsov function that considers flow effects is utilized to compare with phase-field simulation. The boundary layer model in presence of convection is deduced based on the framework of LGK model for diffusion-controlled free dendrite growth^38^. As the temperature is assumed uniform in the system and the undercooling is low, the effects of latent heat and interface attachment kinetics are neglected. The total undercooling is composed of


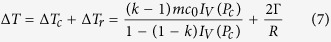


where Δ*T*_*c*_, Δ*T*_*r*_ are solutal and curvature undercooling, respectively. And *R* is dendrite tip radius, *P*_*c*_ is solutal Péclet number, *P*_*c*_ = *RV*/2*D*_*l*_, where *V* is dendrite growth velocity. *I*_*V*_(*P*_*c*_) is Ivantsov function of *P*_*c*_ and *I*_*V*_(*P*_*c*_) = *P*_*c*_exp(*P*_c_)*E*_1_(*P*_*c*_) with *E*_1_ the exponential integral function. The tip radius, *R*, is given by


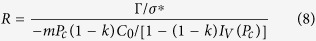


It is assumed that the selection parameter σ^*^ is not influenced by convection (here σ^*^ = 1/4π^2^) in the boundary layer model. In presence of flow, the classical Ivantsov solution for solutal saturation, Ω_*C*_, is replaced with the following modified stagnant film solution


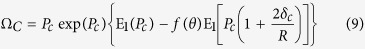


where 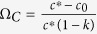, in which c^*^ is solute concentration in the liquid at solid-liquid interface and *δ*_*c*_ is solutal boundary layer thickness^11^


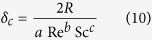


where *Re* is Reynolds number, 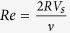, in which *V*_s_ is the instantaneous settling velocity, *Sc* is the Schmidt number, 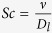, *a* = 0.5773, *b* = 0.6596, *c* = 0.5249. *f(θ*) is an angular factor accounting for the effect of growth directions with respect to gravity on the dendrite tips, and *θ* is the so-called Eulerian angle defined in the same way by Badillo^9^: *θ* = 0° for downward growing tip, *θ* = 90° for perpendicular ones, and *θ* = 180° for upward growing one. Apparently, Eq. (9) resumes to Ivantsov relations when the boundary layer thickness is infinitely large or *f(θ*) is vanished. The exact form of *f(θ*) adopted by Sekerka *et al*.^11^,^34^ is


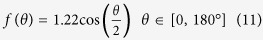


**Figure 6:**
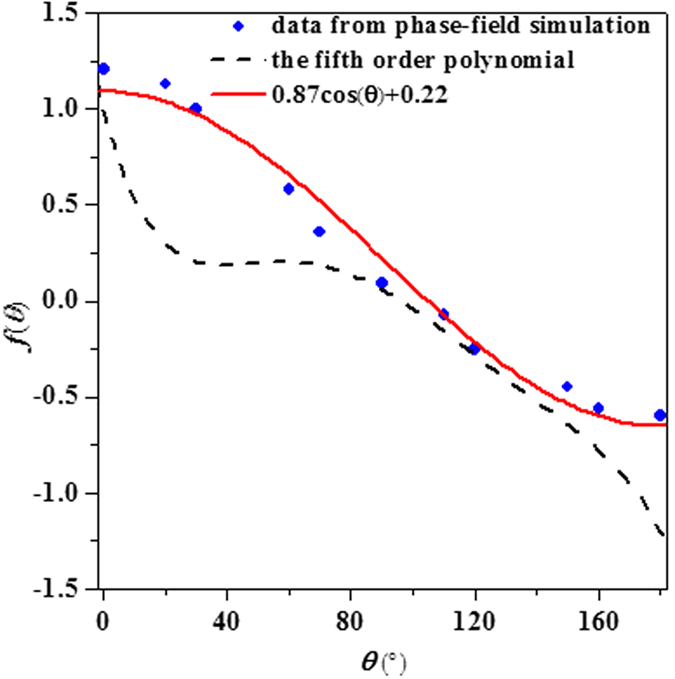
The variation of *f(θ*) obtained from phase-field simulations and the fitted results using the proposed relationship Eq. (12). The fifth order polynomial by Badillo *et al*.^9^ (dashed line) is also imposed for comparison.

**Figure 7:**
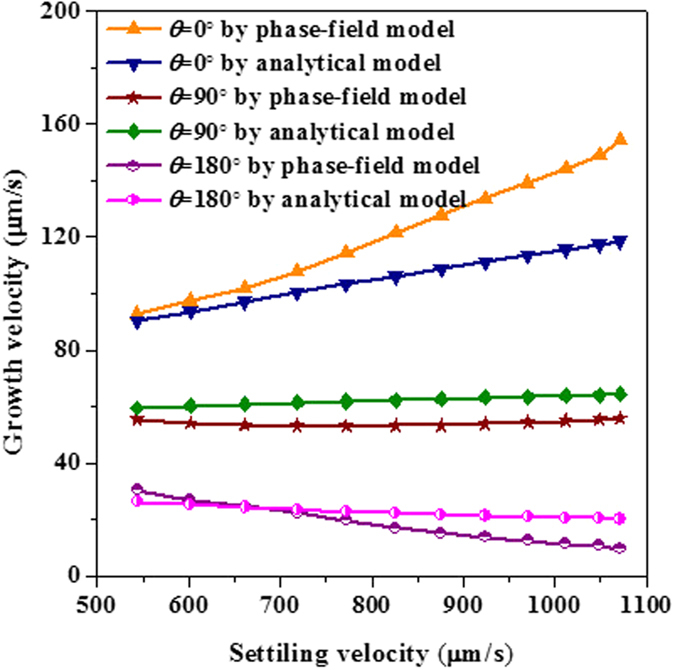
Comparison of tip velocities versus settling velocities between phase-field simulations and prediction by the boundary layer model with *f(θ*) expressed in Eq. (12).

As noted by Badillo *et al*.^9^, the value of *f(θ*) is not negative and it would always result in a greater tip velocity in presence of convection than the diffusion value. To circumvent this irrationality, they fitted the experimental data and obtained a fifth order polynomial^9^. Obviously, the complicate polynomial formulation of *f(θ*) is not theoretically rigorous, hence a simpler expression derived from Eq. (11) is proposed


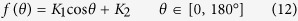


where *K*_1_ is an amplitude parameter controlling the effect of Eulerian angle on the growth dynamics and *K*_2_ is an asymmetry factor, when it is set to 0, it implies that the calculated velocity at *θ* = 90° will have the same value to the diffusional occasion. The original data points of *f(θ*) extracted from phase-field simulation at *t* = 0.51 s when the perpendicular arm growth reaches steady state are shown in Fig. 6, in cases of three different initial orientations (0°, 20°, 60°). The least-squares fitting is adopted to find the optimal values of *K*_1_ and *K*_2_, which are then convinced to be 0.87 and 0.22, respectively, just as the solid line in Fig. 6 illustrates. Plotting of the polynomial^9^ is also imposed in the figure. As expected, the phase-field simulated data deviate a lot from the polynomial curve, in particular at the angle between 20° and 70° where the polynomial curve is almost flattened, physically not reasonable. Figure 7 shows the comparison of the calculated tip velocities versus settling velocities between phase-field simulations and the analytical model. The comparison begins when the tip growth of perpendicular arm arrives at steady state. Generally, with the proposed *f(θ*) of Eq. (12) the analytical predictions shows a good agreement with phase-field simulation, especially at lower settling velocity. The underestimation of growth velocity for the downward growing arm at higher settling velocities may be mainly due to sharply changes of the solute boundary layer ahead of the tip when the dendrite moves very fast.

### Polycrystalline growth in gravity environment

**Figure 8:**
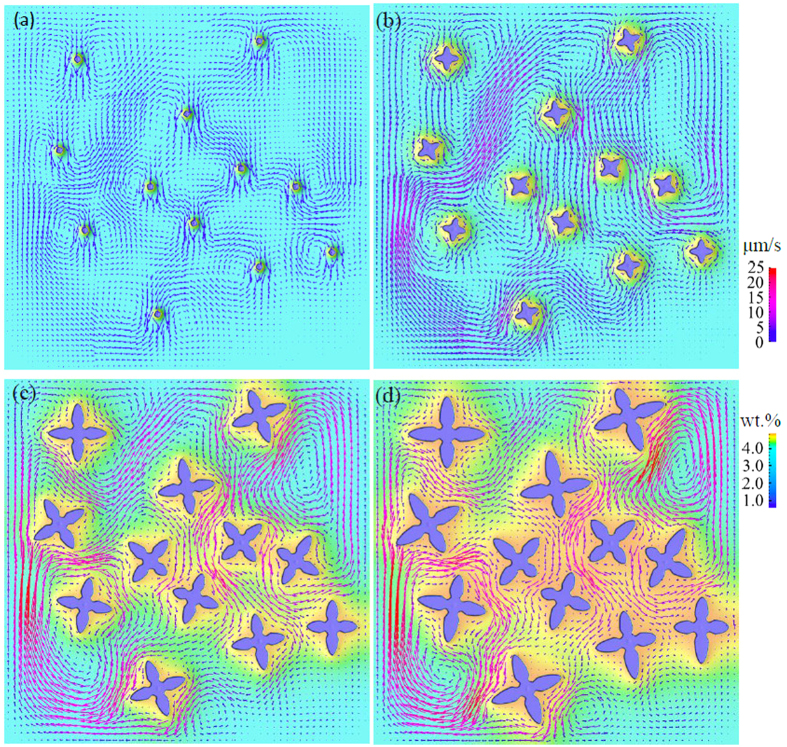
Evolutions of fixed multiple dendritic growth considering solutal convection, (**a**) *t* = 0.02 s, (**b**) *t* = 0.21 s, (**c**) *t* = 0.81 s and (**d**) *t* = 1.21 s. The upper color legend is for flow velocity and the lower for solute concentration. Detailed flow and dendritic growth evolution can be seen in Supplementary Video 1.

**Figure 9:**
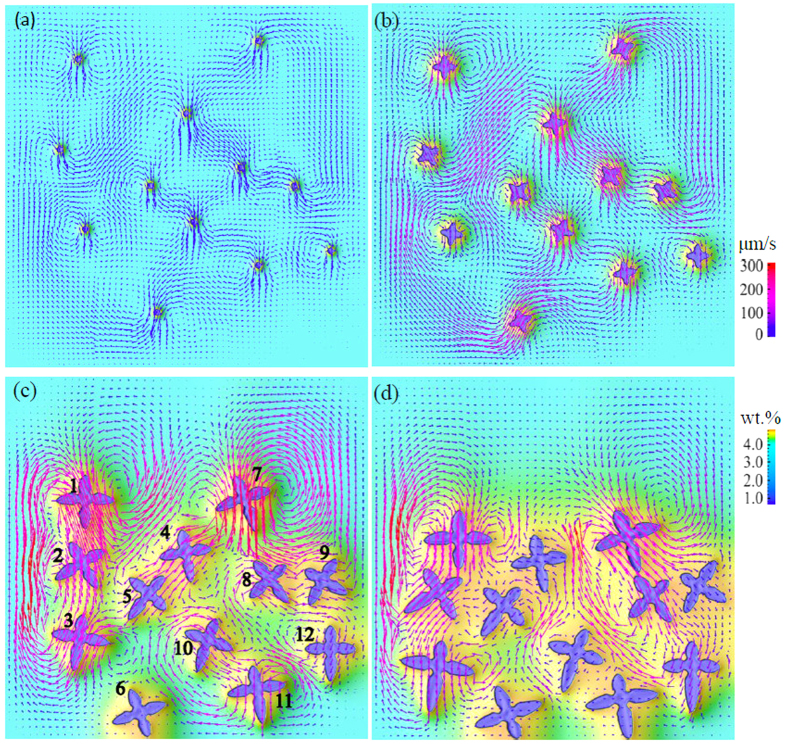
Evolutions of the coupled behaviors of solutal convection, dendritic motion and growth of multiple dendrites using the present model, (**a**) *t* = 0.02 s, (**b**) *t* = 0.21 s, (**c**) *t* = 0.81 s and (**d**) *t* = 1.19 s. The twelve dendrites are numbered in (**c**) for further citation. The upper color legend is for moving velocity and the lower for solute concentration. Detailed evolution of flow, motion and growth of dendrites can be seen in Supplementary Video 2.

The dendritic motion and melt convection during polycrystalline solidification will differ dramatically from that of single crystal growth. The interaction between crystal growth, solutal convection and solid motion becomes more complex because of the impinging effect of multiple dendrites. This results from that when two crystals get close, both the symmetry of solutal boundary and flow pattern which are observed during single free dendrite growth are destroyed. As well to clarify such interacting mechanism during polycrystalline growth, simulations with solutal convection alone and considering both solid and liquid flow are performed as shown in Figs 8 and 9. In simulations, 12 nucleation seeds are randomly placed initially with random orientations in a domain with size of 642 μm × 642 μm. For the simulation considering motion of dendrites, initially, similar to the single crystal growth, all crystals move downwards and a descending melt flow generates around each one, being absence from interactions with others, as shown in Fig. 9(a). As a result, dendrites settle vertically. As crystals growing into larger size, the surrounding flow comes to interact with others. Consequently, the initial symmetric flow vortexes deform and merge with others, inducing strong interdendritic convection, just as illustrated in Fig. 9(b,c). This convergence interrupts the original vertical downward flow, and then imposes an inclined force to the dendrites. Ultimately, at the end of simulation, crystals accumulate at the bottom of the domain, forming a sedimentation zone as described in Fig. 9(d). It may be noticed that during settling of these dendrites, rotational movement is not apparent which can be ascribed to the not sufficient shear force that acts upon the dendrites by the melt flow. When compared with growth of dendrites in Fig. 8, generally, there is no obvious difference of the flow pattern in liquid that can be observed for the two cases at early stage but the lower flow strength in simulation driven by solute buoyancy alone. Interestingly, the ratio of maximum flow velocity between the two cases is around the value of Δ*ρ*_*sl*_/Δ*ρ*_*c*_.

The sedimentation zone constantly occurs in experiments. Beckermann and Wang performed experiments to study the equiaxed dendritic solidification of NH_4_Cl-H_2_O solution in a square cavity^39^. It was observed that the crystals settled down during solidification and gradually formed a sedimentation bed on the cavity floor, which was a zone of negative segregation. This phenomenon is further confirmed and characterized through more delicate experiments^40^,^41^. Both present simulation and these experiments have demonstrated that the motion of crystals due to gravity is common and plays a crucial role on the formation of the macrosegregation pattern. Nevertheless, since the solute is also heavier than the solvent in present case, it also tends to accumulate at the bottom of domain, as indicated by the solute contour in Fig. 9(d). In this situation, the negative segregation resulting from the sedimentation will be reduced to some extent. Also as demonstrated by Wang *et al*.^2^ with the liquid concentration increasing due to solute rejection, the density difference between solid and liquid reduces which would further lighten the macrosegregation. On the contrary, for other alloy systems, such as Fe-C alloy, the lighter rejected solute C floats upwards, while the solidified crystals that are lack of C settle down. In this situation, the negative segregation will be quite severe.

**Figure 10:**
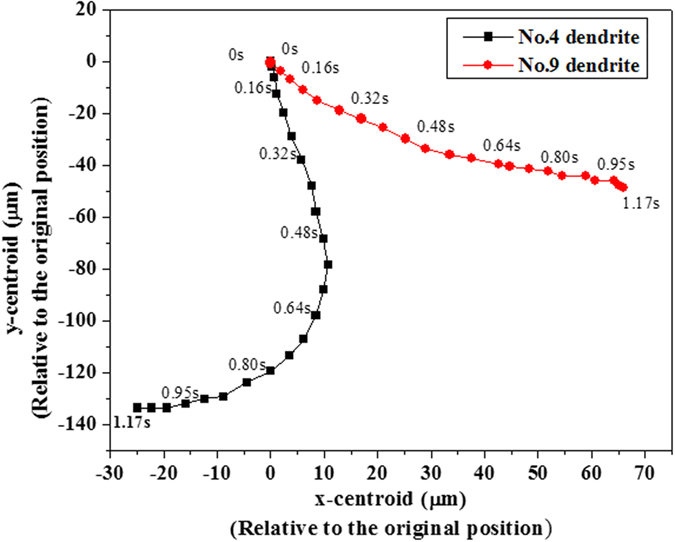
Trajectory of No. 4 and 9 dendrites marked in Fig. 8(c) on the x–y plane. Centroids of the dendrites are plotted relative to their initial position.

**Figure 11:**
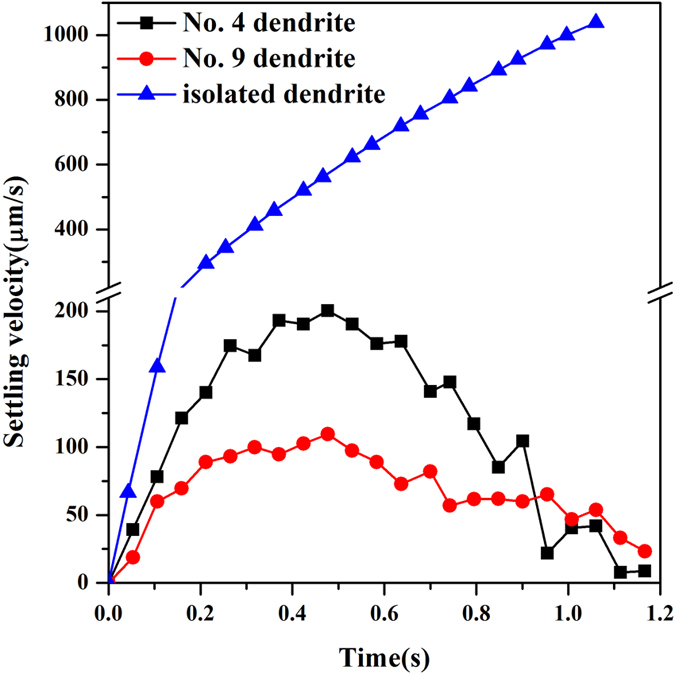
The variation with time of the settling velocity of No. 4 and 9 dendrites, as well as the isolated mobile dendrite in Fig. 1.

In order to clearly demonstrate the motion of solid during polycrystalline solidification, the walking routes of two selected dendrites, No. 4 and 9 as marked in Fig. 9(c), are tracked in Fig. 10. Changes of centroid locations of the two dendrites are recorded relative to their initial positions. From the trajectory, it is clearly observed that No. 4 dendrite firstly tilts to right during its settling, then moves back to left. In contrast, No. 9 dendrite keeps the tendency of tilting to right, and settles down slowly in the vertical direction. These different behaviors of two dendrites are due to their exposure to different flow environments as illustrated by the flow field in Fig. 9. The measured settling velocities of these two dendrites, together with the isolated mobile dendrite in Fig. 1 are plotted in Fig. 11. It can be seen that unlike the linear acceleration of the isolated dendrite, the two dendrites experience an alternation from acceleration to deceleration, and both their absolute velocities are much lower than that of isolated one. This phenomenon demonstrates that during polycrystalline growth, the flow is able to give a lifting force which counteracts the gravity to a dendrite. At the end of simulation, i.e. after 1.1 s, the velocities of the two dendrites tends to 0 due to their arrival at the sedimentation zone. Apparently, dendrites during polycrystalline solidification experience different drag and buoyant forces and thus the overall flow and motion behavior are rather distinct from those of the single dendrite^42^. The walking routes of multiple dendrites as well show obvious sensitivity to the melt flow though the motion depends on the density difference between solid and liquid. These motion behaviors therefore interpret the applied external field (e.g. electromagnetic field) to control microstructure of castings via generating external forces on the solidifying crystals to overwhelm the gravity.

**Figure 12:**
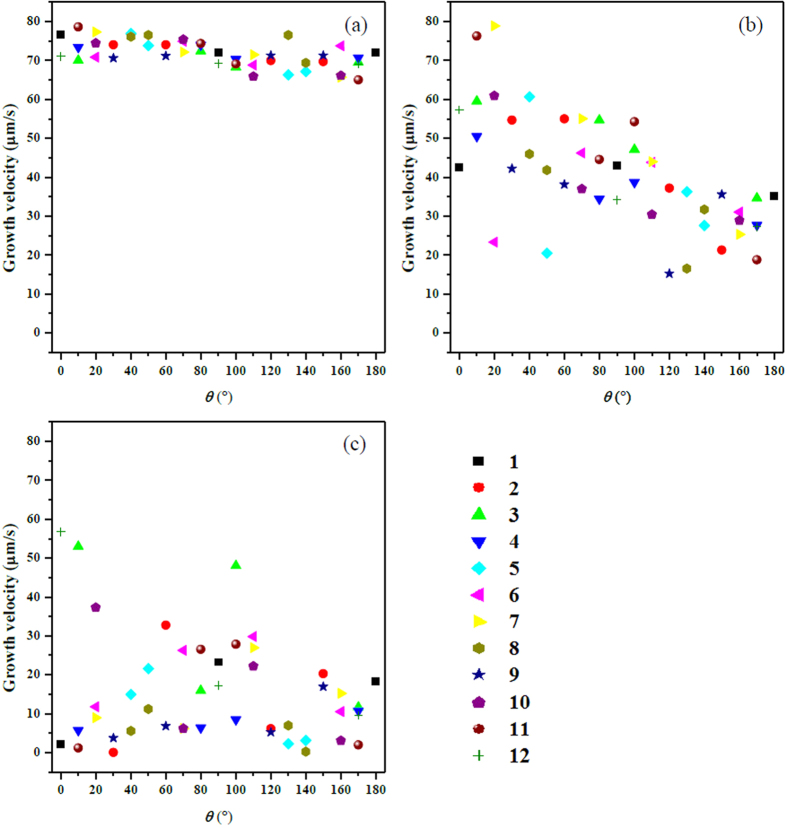
Growth velocities of twelve dendrites at different solidification time: (**a**) 0.21 s (**b**) 0.64 s (**c**) 1.17 s.

The convection and solute distribution around a dendrite during polycrystalline solidification is quite different from that of free isolated dendrite. This is because of the interactions between dendrites, such as the soft-impingement stemmed from the overlapped solute boundary layers^32^,^43^. Dendrites in polycrystalline material usually have random orientations, which results in the uncertainty of the interplay and their growth dynamics. For quantitative illustration of these complexities, the growth velocities of twelve dendrites versus the Eulerian angle are measured in Fig. 12. Conclusions can be drawn from these results as follows. (1) At *t* = 0.21 s, not only arm tips at different directions of one dendrite but also dendrites with different orientations show almost the same growth velocity, approximate to 75 μm/s. This is the typical characteristic of free dendrite growth at the initial stage^32^,^43^, i.e. growth velocities are mainly controlled by diffusion, seldom affected by convection and soft-impingement effects. (2) At *t* = 0.64 s, the solid motion effect dominates the growth. It can be seen that the velocities satisfy the outcome obtained in the above section for single dendrite, i.e., the larger the Eulerian angle is, the more slowly the arm grows. The velocity varies from 15 μm/s to 80 μm/s. (3) At the end of simulation *t* = 1.17 s, the dominant effect switches to soft-impingement effect, since the dendrites at this time almost settle down to the bottom of the domain, and accumulate together to form the deposition zone. The solute piles up heavily between dendrites, which decreases or even eliminates the tip undercooling. In this situation, most dendrites growing slowly and some of them behave against to the convection effect. For instance, the upward growing arm of No. 1 dendrite exhibits a larger velocity than the downward arm.

**Figure 13:**
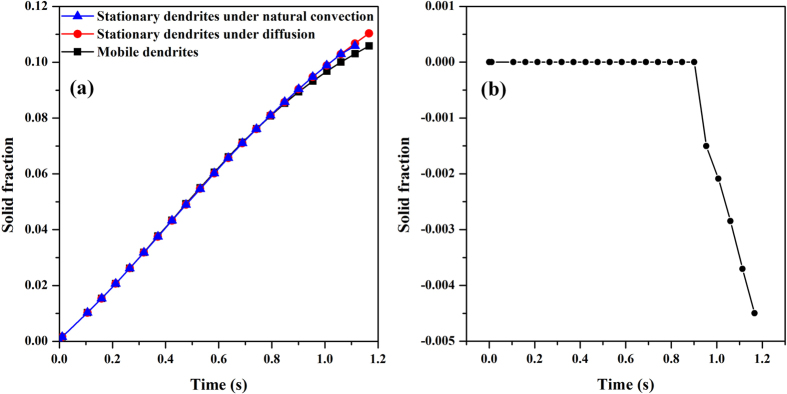
(**a**) Solid fraction as a function of time in three cases: mobile dendrites, stationary dendrites under natural convection, and stationary dendrites under diffusion; (**b**) Difference of solid fraction between mobile dendrites and diffusion-limited stationary dendrites.

However, although the simulations have taken into consideration the effects of solutal convection and solid motion, one interesting phenomenon that the variation of solid fraction with time does not show much visible difference from that of cases under the diffusion-controlled or just considering the solutal convection alone. The simulated dendritic morphologies under pure diffusion can be seen in the Supplementary Materials (Fig. S4). The evolution of solid fraction is shown in Fig. 13(a), and the difference between mobile dendrites and stationary ones with pure-diffusion is plotted in Fig. 13(b). Before *t* = 1.0 s the increment of solid fraction is free from the influence of dendrite motion and solutal convection. Afterwards, the deviation becomes noticeable, appearing as a sharp decrease of the solid fraction of mobile dendrites because of the accumulation of dendrites. This implies that the movement of dendrites accelerates the impinging process which prevents solid from growing further into melt.

## Conclusion

A phase-field model for simulating dendrite-melt two-phase flow during binary alloy solidification is proposed. It aims to uncover the coupling phenomena between melt convection, solid motion, solute diffusion and crystal growth during solidification. Compared to previously used phase-field Lattice Boltzmann method, it is not necessary to track explicitly the interfaces between different phases or different grains to resolve the motion of crystals, but still able to predict the rotational and translational motion of a solid with complex morphology in the proposed method. Simulations of a single dendrite settling down under gravity demonstrate that the motion of solid exerts crucial effects on the dendritic growth morphology. The solid motion promotes the downstream arm growth rather than the upstream arm, which is contrary to the case under only melt convection. With the simulations of single dendritic growth at different orientations and previous experimental data^9^, the analytical boundary layer model that predicts the effects flow on dendritic growth dynamics is modified to well relate the dendritic tip velocities with respect to flow directions. A generally good agreement is achieved between phase-field simulation considering dendrite motion and the analytical solution. The proposed phase-field model incorporating two-phase flow is then extended to tackle the complex solidification phenomenon of multiple crystals. Because of dendrites motion, a sedimentation zone of crystals is observed in the simulation, which reveals the fundamental process of such phenomenon often occurred in industrial ingots. The growth dynamics, movement path and solid fraction of polycrystalline solidification are characterized in detail which uncovers the interacting process between solid motion, melt flow and crystals growth. These interactions closely relate to the stochastic factors existing in solidification, such as the amount of seeds, relative position of nucleating sites and crystallographic orientations. And hence the growth and movement of each dendrite not only depends on its own buoyant force and local solute boundary layer, but also associates to the long-range melt flow.

The extension of the model to three dimension is direct since all the coupled physical models have already been well developed separately in 3D. The simulations presented in this work are for melt flow with small Reynolds number, numerical analysis and benchmark simulations are necessitated to verify the possibility in coping with solid particle moving and rotating at high speed or flow with large Reynolds number. In addition, direct collision of dendrites in this model is neglected. The parameters in the polycrystalline phase-field model are given artificially and not derived from material properties, which makes the polycrystalline growth can not be closely related to real materials and compared with *in situ* experiments quantitatively^32^,^43^. This deficiency may be solved using the front tracking method to calculate the orientation proposed in the ref. 44 when the grain boundary energy and rotation change of crystal orientation are included into their model.

## Additional Information

**How to cite this article:** Qi, X. B. *et al*. Modeling of coupled motion and growth interaction of equiaxed dendritic crystals in a binary alloy during solidification. *Sci. Rep.* **7**, 45770; doi: 10.1038/srep45770 (2017).

**Publisher's note:** Springer Nature remains neutral with regard to jurisdictional claims in published maps and institutional affiliations.

## Supplementary Material

Supplementary Information

Supplementary Video 1

Supplementary Video 2

## Figures and Tables

**Table 1:** Physico-chemical parameters of Al-Cu alloy and applied solidification parameters in numerical simulations^2^,^45^.

Melting point of Al, *T*_*M*_	934.00 K
Solute partition coefficient, *k*	0.14
Initial concentration, *c*_0_	4 wt.% Cu
Liquidus slope, *m*	−2.65
Solute diffusion coefficient in liquid, *D*_*l*_	2.4 × 10^−5^ cm^2^/s
Gibbs-Thomson coefficient, *Γ*	2.36 × 10^−5^ cm·K
Surface energy anisotropy strength, *ε*_*4*_	0.05
Interface width parameter, *ξ*	10
Kinematic viscosity of the liquid phase, ν_*l*_	5.0 × 10^−3^ cm^2^/s
Kinematic viscosity of the solid phase, *ν*_*s*_	5.0 × 10^3^ cm^2^/s
Density of the liquid phase, *ρ*_*l*_	2.45 g/cm^3^
Density of the solid phase, *ρ*_*s*_	2.7 g/cm^3^
Solutal expansion coefficient, *α*_*s*_	9.2 × 10^−3^/wt.%
Gravitational acceleration, *g*	−1000 cm/s^2^
Initial radius of the seed, *r*_0_	2.14 μm
Dimensionless solute super-saturation, *Ω*	0.2
Dimensionless time step, Δ*t/τ*	0.05
Dimensionless minimum mesh spacing, Δ*x/W*_0_	~0.6
