# Supplementary material for: Modeling of coupled motion and growth interaction of equiaxed dendritic crystals in a binary alloy during solidification
Source: Sci Rep. 2017 Mar 31;7:45770. doi: 10.1038/srep45770 (PMC5374713; doi:10.1038/srep45770)
Supplement: Supplementary Information [file srep45770-s1.pdf]

Supplementary information

Modeling of coupled motion and growth interaction of equiaxed dendritic crystals in a binary alloy during solidification

Xin Bo Qi, Yun Chen*, Xiu Hong Kang, Dian Zhong Li, Tong Zhao Gong

Shenyang National Laboratory for Materials Science, Institute of Metal Research, Chinese Academy of Sciences, Shenyang, Liaoning, 110016 P. R. China

*chenyun@imr.ac.cn

Numerical tests of the model

Rising of a circular particle in bulk liquid

Before applying the proposed phase-field model to the case of moving dendrites, it is necessary to validate the model in handling solid-liquid flow in some simple situations that can be benchmarked using analytical solutions or reported numerical simulations. The convergence of the model is studied by simulating a freely floating solid particle in two dimensions. The particle is assumed to be circular with radius a equal to $400d_0$ (here, $d_0 = 1.96\times10^{-6}$ cm). The size of computational domain is $250a \times 250a$, which is to guarantee the particle moving freely in bulk liquid and not affected by the wall. The densities of two phases are ρ_s = 2.45 g/cm^3 and ρ_l = 4.9 g/cm^3, respectively. Given that $\rho_l / \rho_s = 2$ and density variation is not sufficiently small, the averaged density $(\rho_l + \rho_s)/2$, instead of pure liquid density, is selected as the background density in the Boussinesq approximation [1]. The kinematic viscosities of liquid and solid are chosen to be 5.0×10^{-3} cm^2/s and 5.0×10^{3} cm^2/s, respectively. The value of W_0 ranges from $5d_0$ to $40d_0$. The simulated rising particle with $W_0 = 10d_0$ is presented in Fig. S1. From the distribution of phase-field variable evolving with time in Fig. S1(a), it can be seen that though the particle is not assumed to be rigid, it always keeps its initial shape and size during rising, as well no stretch and deformation are observed. The closer flow field depicted in Fig. S1(b) shows each point in the particle region moves upwards at a same speed. Two vortexes generate symmetrically on both sides of the particle as a result of flotation in Fig. S1(c, d). These features of the moving particle imply that the proposed model is able to

correctly simulate the solid particle motion in the liquid.

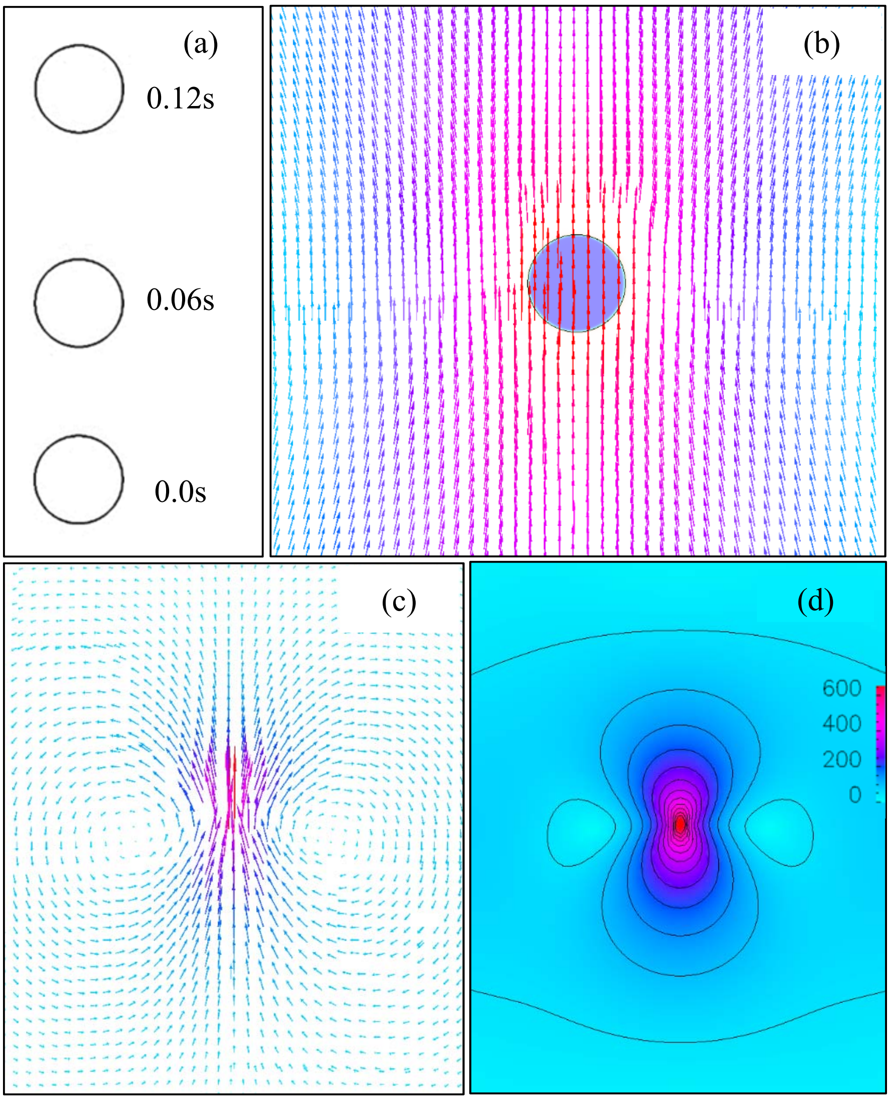

Fig. S1 Simulation of a rising circular particle in bulk liquid: (a) time evolution of the particle shape where the solid line represents $\phi = 0$; (b) closer flow field in and around the particle; (c) the flow field and (d) its nephogram from a bird's-eye view, the interval between velocity isolines is 50 μm/s.

Since the position of solid phase is updated by the advection term in the phase-field equation, a test of the choice of W_0 is provided. The variation of the floating velocity with time for different choices of W_0 is plotted in Fig. S2. Initially, the particle rises slowly but with the highest acceleration. Due to the viscous friction that is proportional to the square of the rising velocity, the acceleration decreases with the increment of velocity. Eventually, the particle reaches steady state where the driving

force is balanced by frictional force. For all the choices of W_0, the rising velocities evolve with the same trend but just differ at the steady state. The drag force acting upon a circular particle by the liquid has been proposed by Van Dyke [2]

$$F_d = C_D \rho_l V^2 a = \frac{4\pi\rho_l \nu V}{\ln\left(\frac{4\nu}{aV}\right) - \gamma - \frac{1}{2}} \tag{1}$$

where C_D is drag coefficient, $\gamma = 0.5772$ is Euler constant, V is rising velocity of the particle, ν is kinematic viscosity of the fluid. Terminal velocity V_{inf} is achieved when the sum of gravity and drag force is equal in magnitude to buoyance, and yields

$$V_{inf} = \frac{(\rho_l - \rho_s) a^2 g [\ln(\frac{4\nu}{aV_{inf}}) - \gamma - \frac{1}{2}]}{4\rho_l \nu} \tag{2}$$

where $g = 980$ cm/s^2 is gravitational acceleration. After substituting the parameters of two phases into Eq. (2), $V_{inf} = 0.072$ cm/s is calculated through an iterative algorithm. Comparison of phase-field simulated floating velocity of a particle with the analytical solution is illustrated in Fig. S2. As the interface width parameter used in the simulation decreases, the prediction by the phase-field model shows convergence and approaches the analytical solution. In following simulations, the interface width parameter W_0 is chosen to be $10d_0$ in consideration of both numerical accuracy and computational efficiency.

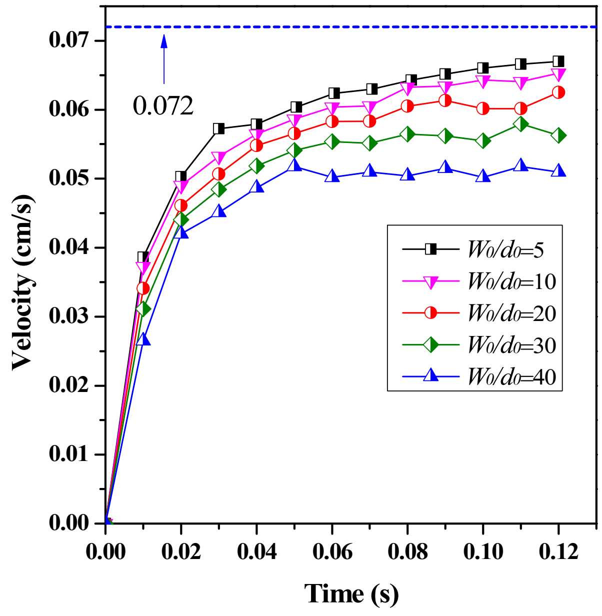

Fig. S2 The simulated time evolution of the rising velocity of a circular particle in bulk liquid and comparison with the analytical solution.

Rotation of a growing dendrite at a constant angular velocity

The rotation of a fourfold symmetry dendrite growing from undercooled melt is simulated, aiming to test the advection and curl terms in the crystallographic orientation equation. All of the computational parameters for dendrite growth are identical to that proposed by Karma [3]. An angular velocity which is set to be $\pi/(10000\,\tau_0)$ is imposed over the entire computational domain. Initially, the orientation of the dendrite is 0°. After a 45° rotation under the given angular velocity, the simulated dendritic morphology and the imposed rotational velocity field are presented in Fig. S3(a). Generally, the dendrite keeps the fourfold symmetry, as it should be. Nevertheless, when the crystallographic orientation change resulting from rotation is canceled in the phase-field equation, a distorted dendrite is obtained in Fig. S3(b), which is similar to the swirling dendrite obtained by Yamaguchi [4]. In order to examine the change of orientation quantitatively, time evolution of orientation and the

targeted angle calculated from the prescribed angular velocity are plotted for comparison in Fig. S3(c). A wonderful agreement is achieved, which claims the accuracy of the proposed model in handling the crystallographic orientation evolution. Further quantitative comparison of the tip growth velocity with pure diffusion-controlled case and that by Karma [3] is plotted in Fig. S3(d). Since the flow velocity is always perpendicular to the growth direction, the rotation does not show pronounced influence on the dendritic growth dynamics, as indicated by the coincided three curves. This test clarifies the capability of present model to tackle the rotational problem of dendritic growth, and indicates that the treatment of orientation in response to liquid flow is able to describe the orientation change physically.

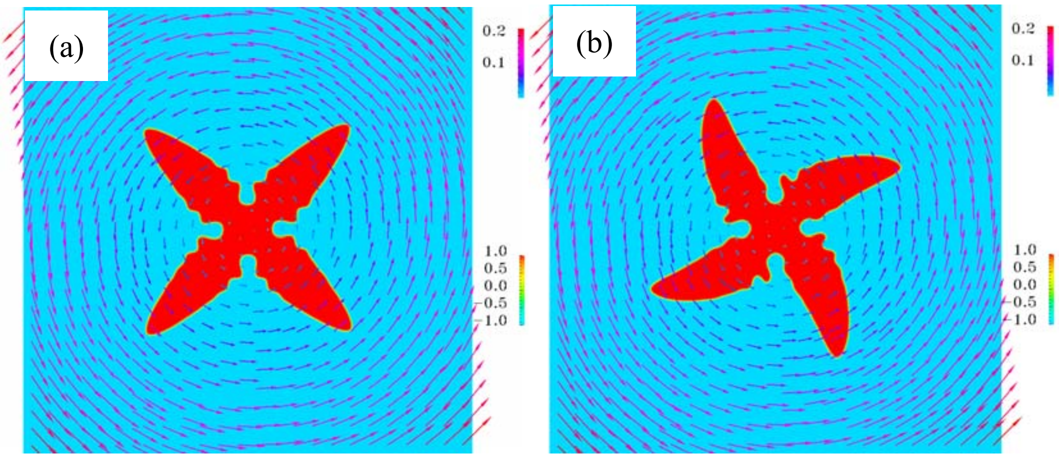

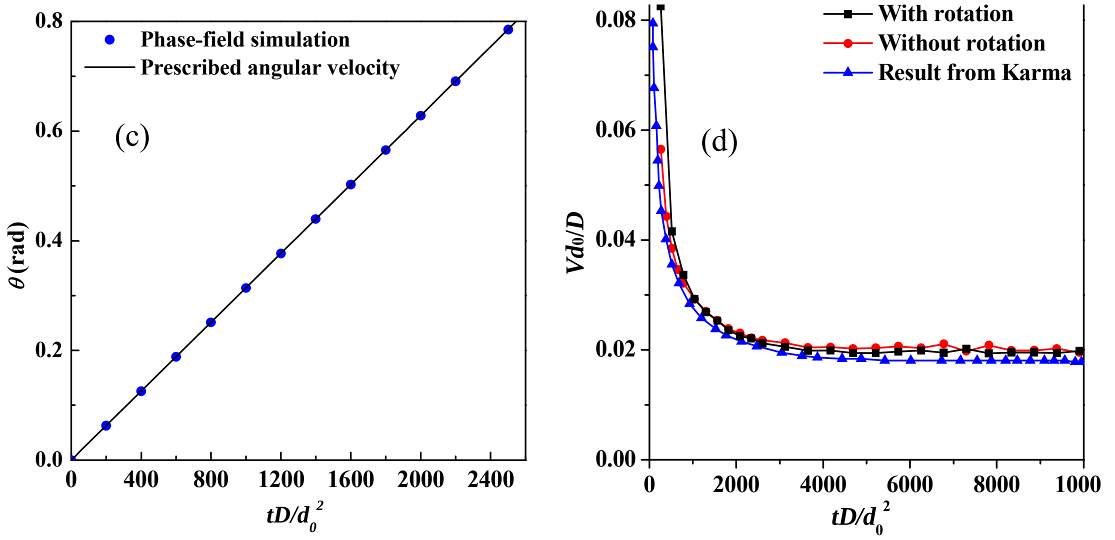

Fig. S3 Phase-field simulation of dendritic solidification with an imposed rotational velocity field. (a) The simulated dendrite after a 45° evolution. (b) Disorted dendrite resulting from the cancel of the crystallographic orientation change in the phase-field equation. (c) Comparison of time evolution of orientation with the targeted angle calculated using the prescribed angular velocity. (d) Comparison of the tip growth velocity as a function of time between dendrite growth with rotation, without rotation and data obtained by Karma [3].

Dendritic growth in a shear flow

Simulation of a dendrite immersed in a shear flow is conducted here. In order to make the dendrite rotate and translate simultaneously, different flow velocities V_0 are set at the top and bottom walls, which are $0.2W_0/\tau_0$ and 0, respectively. The computational domain size is $W = 4000W_0$ and $H = 2000W_0$ in the x and y directions, respectively. Dirichlet boundary condition $V=(y/H)*V_0$ is set at the inflow and outflow boundaries. The initial orientation of the seed is 0. Fig.S4 shows the counters of the phase field φ=0 at four different times (t/τ_0 = 100, 1000, 3000, 6000). As can be seen, due to different forces impacted on the upward and downward growing arms, the grain rotates under the action of the flow.

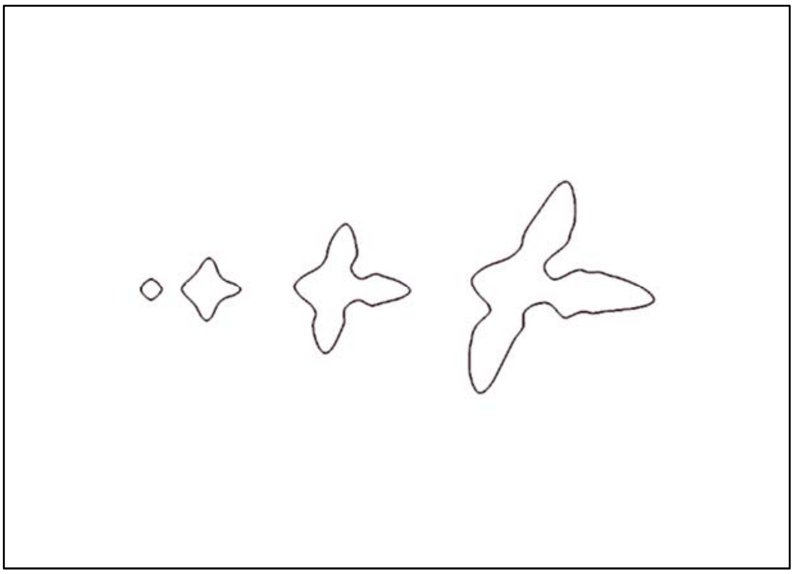

Fig. S4 Counters of $\varphi = 0$ of a dendrite under shear flow at four different times (t/τ_0 = 100, 1000, 3000, 6000).

The time dependence of the grain orientation is shown in Fig. S5. It exhibits an approximate linear reduction, which means that the dendrite rotates clockwise in a constant angular velocity.

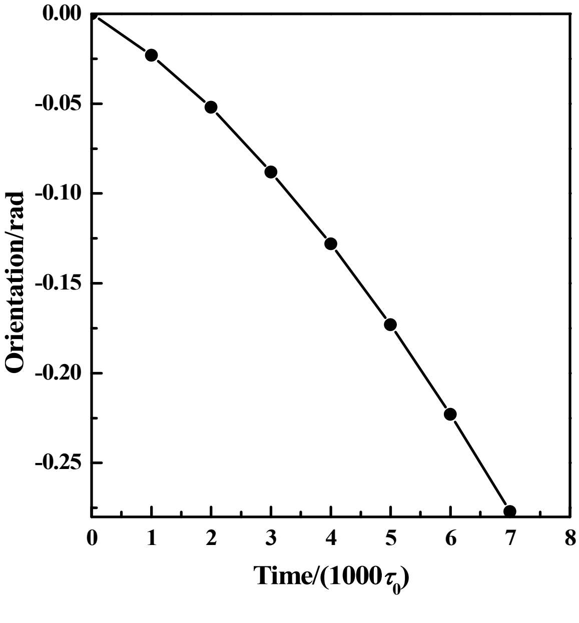

Fig. S5 Time dependence of the grain orientation in a shear flow.

Polycrystalline dendrite growth under diffusion and consideration of solutal convection

In order to present a clear understand of solid motion on dendrite growth, the

polycrystalline growth under diffusion was simulated for comparison. Snapshots of the simulated dendritic morphologies are shown in Fig. S6. From the figures it can be seen that the polycrystalline morphology does not show apparent difference from that with consideration of melt convection alone.

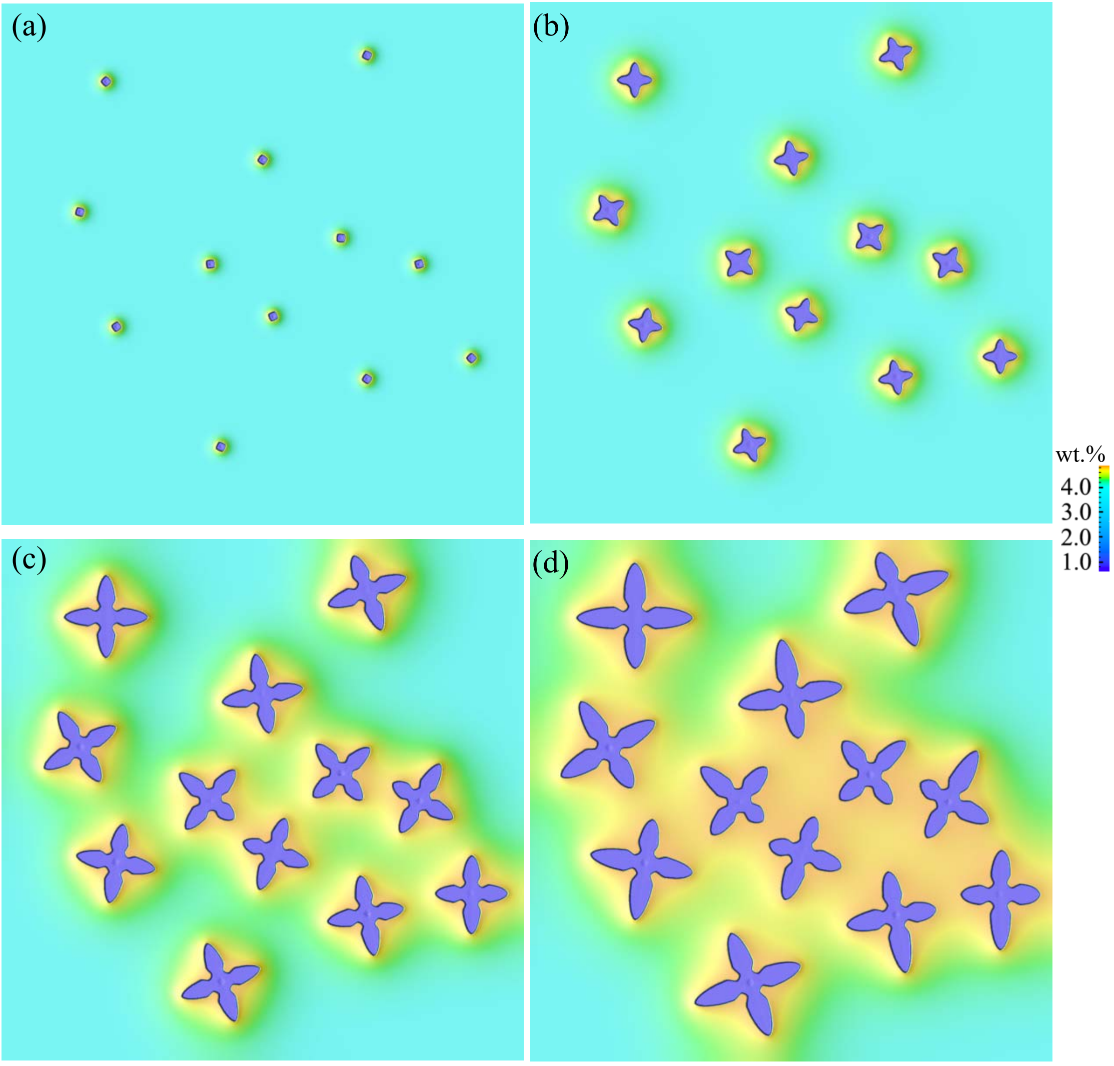

Fig. S6 Evolutions of multiple dendrites without consideration of solutal convection using the present polycrystalline phase-field model. (a) $t = 0.02$ s, (b) $t = 0.32$ s, (c) $t = 0.81$ s and (d) $t = 1.21$ s.

Supplementary video legends

Supplementary Video 1: Dendrites growth with only melt convection

This video shows the convection pattern driven by solutal buoyancy alone and its evolution with time during polycrystalline solidification.

Supplementary Video 2: Dendrites growth with solid motion and melt convection

This video shows the evolution with time of melt convection and movement of

dendrites during polycrystalline solidification, as well as the resulted solute segregation.

References

1. Lee, H. & Kim, J. A comparison study of the Boussinesq and the variable density models on buoyancy-driven flows. *J. Eng. Math.* **75**, 15-27 (2012).
2. Van Dyke, M. *Perturbation methods in fluid mechanics*. 164 (The Parabolic Press, 1975).
3. Karma, A. Phase-field formulation for quantitative modeling of alloy solidification. *Phys. Rev. Lett.* **87**, 115701 (2001).
4. Yamaguchi, M. & Beckermann, C. Simulation of solid deformation during solidification: Compression of a single dendrite. *Acta Mater.* **61**, 4053-4065 (2013).
